# Assessing biodiversity and endemism using phylogenetic methods across multiple taxonomic groups

**DOI:** 10.1002/ece3.1747

**Published:** 2015-10-22

**Authors:** Carlos E. González‐Orozco, Brent D. Mishler, Joseph T. Miller, Shawn W. Laffan, Nunzio Knerr, Peter Unmack, Arthur Georges, Andrew H. Thornhill, Dan F. Rosauer, Bernd Gruber

**Affiliations:** ^1^ Institute for Applied Ecology and Collaborative Research Network for Murray‐Darling Basin Futures University of Canberra Canberra Australian Capital Territory 2601 Australia; ^2^ University and Jepson Herbaria Department of Integrative Biology University of California Berkeley California 94720‐2465; ^3^ Centre for Australian National Biodiversity Research CSIRO Plant Industry GPO Box 1600 Canberra Australian Capital Territory 2601 Australia; ^4^ Division of Environmental Biology National Science Foundation Arlington Virginia 22230; ^5^ Centre for Ecosystem Science School of Biological, Earth and Environmental Sciences University of New South Wales Kensington New South Wales 2052; ^6^ Division of Ecology, Evolution and Genetics Australian National University Canberra Australian Capital Territory 0200 Australia; ^7^ Australian Tropical Herbarium James Cook University Cairns QLD 4870 Australia

**Keywords:** Australia, biogeography, conservation, Murray–Darling basin, phylogenetic diversity, phylogenetic endemism

## Abstract

Identifying geographical areas with the greatest representation of the tree of life is an important goal for the management and conservation of biodiversity. While there are methods available for using a single phylogenetic tree to assess spatial patterns of biodiversity, there has been limited exploration of how separate phylogenies from multiple taxonomic groups can be used jointly to map diversity and endemism. Here, we demonstrate how to apply different phylogenetic approaches to assess biodiversity across multiple taxonomic groups. We map spatial patterns of phylogenetic diversity/endemism to identify concordant areas with the greatest representation of biodiversity across multiple taxa and demonstrate the approach by applying it to the Murray–Darling basin region of southeastern Australia. The areas with significant centers of phylogenetic diversity and endemism were distributed differently for the five taxonomic groups studied (plant genera, fish, tree frogs, acacias, and eucalypts); no strong shared patterns across all five groups emerged. However, congruence was apparent between some groups in some parts of the basin. The northern region of the basin emerges from the analysis as a priority area for future conservation initiatives focused on eucalypts and tree frogs. The southern region is particularly important for conservation of the evolutionary heritage of plants and fishes.

## Introduction

Accurate assessment of native biodiversity is required to effectively manage and conserve areas of high value (Ferrier 2002; Pressey et al. 2013). One question that remains unclear is which diversity metrics are the most accurate and efficient for identifying underlying biodiversity patterns. Such metrics ideally should encompass both species richness and compositional distinctiveness, the latter often measured as phylogenetic diversity (Faith 1992; Diniz‐Filho et al. 2013). Mapping species and phylodiversity provides useful insights (Wiens and Donoghue 2004; Rodrigues et al. 2005), but agreement on the most effective approach to assess biodiversity comprehensively in a single study remains elusive. In particular, approaches for assessing concordance in spatial patterns of biodiversity across multiple taxa are not yet well developed. Few case studies addressing multitaxon patterns of phylogenetic diversity have been attempted (Sobral et al. 2014; Zupan et al. 2014). Here, we investigate the use of several diversity metrics simultaneously and more specifically emphasize phylogenetic endemism approaches (Rosauer et al. 2009; Mishler et al. 2014).

Numerous case studies have demonstrated the value of the phylogenetic diversity (PD) index for providing a more satisfactory assessment of biodiversity (Faith 1992; Forest et al. 2007; Diniz‐Filho et al. 2013). Areas of high importance for conservation, which were not identified by traditional metrics based on species richness alone, emerge clearly from analyses based on new phylogenetic methods (Mishler et al. 2014). PD has been used to study taxonomic groups separately (Rodrigues et al. 2005; Rosauer et al. 2009), but the greatest value from these new approaches is obtained when spatial patterns of biodiversity are concordant across multiple taxa. The idea of combining multiple studies at global and regional scales shows promise (Gaston et al. 1995; Adams 2008; Thuiller et al. 2011; Jansson et al. 2013), but such studies are often correlative, lack a substantive spatial component, or focus mainly on species diversity (Heino 2010; Barreto de Andrade et al. 2014). Some case studies addressing multitaxon patterns of biodiversity have been attempted with varying success. Schuldt and Assmann (2010) conducted a multitaxon analysis of 12 invertebrate groups including mammals and vascular plants, across Europe. Partial correlations and PCA of species richness and endemism were used across taxa to identify areas with high taxonomic and phylogenetic diversity (Tucker and Cadotte 2013). In another study (Crisp et al. 1995), concordance of spatial patterns across multiple taxa of angiosperms in Australia was demonstrated using traditional cladistic biogeographical methods. Statistical significance of concordant richness across taxonomic levels has been used as a basis to identify indicator groups as surrogates of overall diversity (Palitzsch and Rahbek 2002; Lovell et al. 2007; Gioria et al. 2011; Fattorini et al. 2012), acknowledging that spatial patterns in one taxonomic group can be informative about spatial patterns of another.

Concordant patterns in phylodiversity can be used for identifying areas that can be considered diversity hubs for conservation (Laity et al. 2015). These methods are useful to ensure that management is directed appropriately in achieving adequate regional or national representation of biodiversity. Compositional dissimilarity is a concept that has been applied to map biodiversity (Belbin et al. 1992), but, until recently (González‐Orozco et al. 2014b), there was no multiple taxon‐based approach that exclusively mapped multiple taxonomic groups based on a large proportion of the Australian flora. These more recent analyses reduced subjectivity but did not include phylogenetic relationships, nor were they applied to smaller regional scales. Failing to identify regions of important phylogenetic diversity at different geographical scales means that strategic decisions in conservation are founded on incomplete information. For example, while Mishler et al. (2014) reported centers of phylogenetic endemism for Australian *Acacia*, we still have limited information on how robust these centers will be in multitaxon analyses. No taxon can be managed in isolation and concurrence in patterns of distribution across the landscape is important for conservation prioritization, so there is strong justification to focus efforts on further developing phylogenetically based assessments across multiple taxonomic groups, in particular in environments surrounded by rapidly expanding agricultural areas, which increases the risk of biodiversity loss.

**Figure 1:**
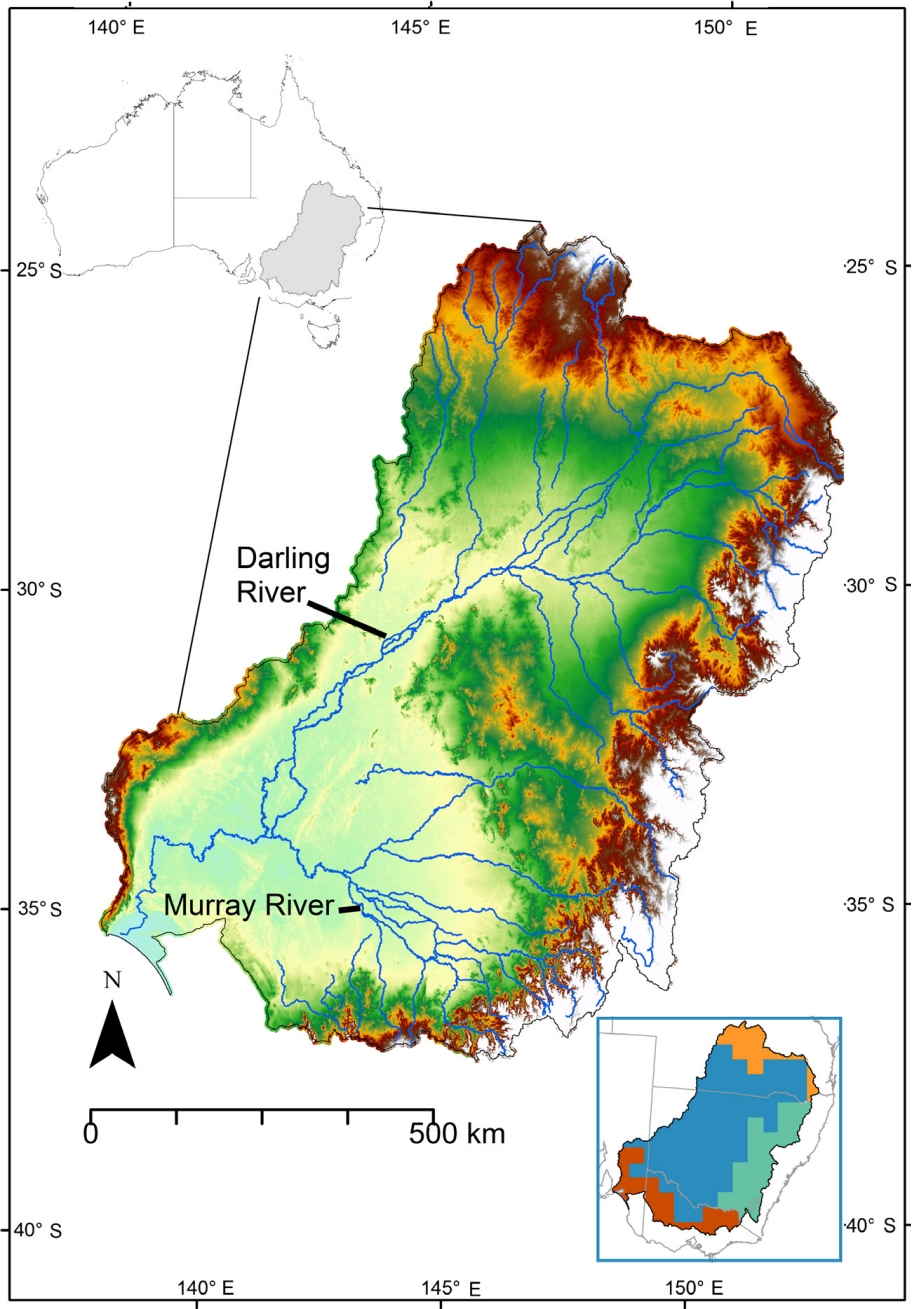
The Murray Darling basin is in southeastern Australia and is approximately one million km^2^ in area. White shades in map indicate high elevations; red and green mid, elevation; and yellow and cyan, lowlands. The box on the bottom right shows the phyto‐geographical regions of the MDB based on a multiple‐species continent‐wide flora regionalization (González‐Orozco et al. 2014b).

The Murray–Darling drainage basin (MDB) is located in southeast Australia, and with an area of 1,061,469 km^2^ is one of the largest river systems in the world; it is also one of the driest (Fig. 1). The MDB has an elevation gradient ranging from 0 to 2228 m. Highest precipitation occurs on the southeast ranges which also experience a cooler temperate climate. Relatively high precipitation (1000–1200 mm/year) also occurs in the north (southern Queensland) which experiences a subtropical climate. The western part of the MDB is part of the semi‐arid interzone of central Australia. The Murray and Darling rivers flow from east to west, fed by multiple tributaries on the eastern uplands. Low relief in parts of the MDB is often represented by sedimentary basins with numerous seasonally ephemeral wetlands, whereas the mountainous areas form part of the Great Dividing Range (GDR) that extends the entire length of the Australian east coast.

Over 200 years of intensive human modification of the basin's landscapes has had a major impact on biodiversity (Koehn and Lintermans 2012). High vulnerability of its environment owing to human pressure (i.e., expansion of agriculture and pastoralism) and the prevalence of threatened species render the MDB an ideal place to apply new techniques for identifying biodiversity hotspots and priority areas for conservation. Research in the MDB has generated a robust body of scientific literature on topics such as water allocation and management, agriculture, climate change, food security, and human settlements (CSIRO 2008; Davies et al. 2010; MDBA 2011; Ashcroft et al. 2013; Head et al. 2013; Koehn 2013; Mills et al. 2013; Saintilan et al. 2013). Plant diversity of the MDB falls into four subregions (see box in Fig. 1), as defined by a continent‐wide multiple species bioregionalization study (González‐Orozco et al. 2014b), but no analyses incorporating phylogenetic methods across multiple taxon groups have been applied to the MDB.

Here, we applied phylogenetic approaches to identify concurrence of spatial patterns of phylogenetic diversity and endemism across five taxonomic groups in the MDB. Our specific objectives were to (1) map spatial patterns of diversity and endemism within the MDB; (2) identify significant centers of diversity and endemism across multiple taxonomic groups within the MDB. The main research question was as follows: Are phylogenetic diversity and endemism spatially concordant across multiple taxonomic groups?

## Materials and Methods

### Taxonomic groups and spatial datasets

Three plant groups (*Acacia*, eucalypts, and all plant genera) and two animal groups (tree frogs and fishes) were included in this study and are hereafter referred to as the test groups. The taxonomic datasets used here were selected because they were readily available and also because they represented both terrestrial and aquatic environments. The spatial datasets containing records for all test groups, projected into an Albers conic conformal coordinate system (code EPSG 3577), were imported into biodiverse 0.18 (Laffan et al. 2010) and aggregated to 25 km × 25 km grid cells. The total numbers of grid cells containing observation records were *Acacia* (1570), eucalypts (1442), plant genera (1791), frogs (872), and fishes (446).

#### *Acacia* and eucalypts

*Acacia* and eucalypts are iconic Australian plant groups. *Acacia* is the most species‐rich genus in Australia with more than 1000 species (Maslin et al. 2003), and there is detailed knowledge of its phylogenetic history, divergence dates (Miller et al. 2013), and excellent data on the distribution of the species in this diverse group (González‐Orozco et al. 2011). Eucalypts are the most common canopy trees in Australia. In this study, we defined the eucalypts as the three genera *Angophora, Corymbia,* and *Eucalyptus* in the Myrtaceae tribe Eucalypteae (Brooker et al. 2006). Spatial information for *Acacia* and eucalypt specimens collected in the MDB were extracted from *Australia's Virtual Herbarium* (AVH 2013; CHAH 2010a). We excluded subspecies from the plant groups and used only the accepted species names from the *Australian Plant Census* (CHAH 2010b). A total of 32,507 records of 279 *Acacia* species and 36,207 records of 248 eucalypt species known to occur within the MDB were used for the analyses.

#### All plant genera

A genus‐level dataset comprising 737 genera and 442,700 records for all vascular plants known to occur in the MDB was used for the analyses. *Acacia*,* Angophora*,* Corymbia*, and *Eucalyptus* were each represented in this dataset by a single terminal branch on the tree. The plant genus dataset was compiled using a checklist of the plants of the lower Murray River, originally gathered by the CSIRO Water Assessment Audit of 2003. Spatial records from the Australia's Virtual Herbarium (AVH 2013) for each genus present in the CSIRO dataset and within the MDB region were extracted from the spatial portal of the Atlas of Living Australia (ALA; http://www.ala.org.au).

#### Frogs

The tree frogs (Hylidae subfamily Pelodryadinae) were used to represent animals that occupy semi‐aquatic environments. A total of 82,686 frog records comprising 42 species for the MDB were used for the analyses. There are 87 described species of tree frogs in Australia (Anstis 2013). The spatial records were compiled as described in Rosauer et al. (2009, 2014). The hylids have a continent‐wide distribution with centers of species richness and endemism in the wet tropics and the Border Ranges between Queensland and New South Wales (Slatyer et al. 2007).

#### Fishes

An MDB fish dataset was compiled to represent aquatic organisms. A total of 8374 records were sourced from the Sustainable Rivers Audit (Davies et al. 2010) representing 22 fish species that occur in the MDB. The present‐day MDB fish diversity is thought to be historically influenced by surrounding regions with the largest proportion of species being shared with southeast Queensland coastal drainages (Unmack 2013). The fish species of the MDB are represented as one single biogeographical province of the Australia‐wide fish bioregions (Unmack 2001).

### Phylogenetic trees

The five phylogenetic trees are shown in Appendix S1. The phylogenetic trees of *Acacia* and eucalypts were pruned from continent‐wide trees (Miller et al. 2013; Mishler et al. 2014) using biodiverse 0.18 (Laffan et al. 2010), to include only MDB taxa. Both the *Acacia* and eucalypt continental phylogenies were generated using a partitioned alignment and the Black‐box tool in RAxML (Stamatakis 2014) in the online CIPRES portal (http://www.phylo.org/index.php; Miller et al. 2010). The MDB phylogenies consisted of 279 *Acacia* species (of 1020 total species reported for Australia; CHAH 2010a; González‐Orozco et al. 2011, 2013) and 248 eucalypt species (of 795 total species reported for Australia; Brooker 2000; González‐Orozco et al. 2014a,b). These represent approximately 25% of Australia's *Acacia* diversity and 31% of Australia's eucalypt diversity. The genus‐level plant phylogeny was also generated using RAxML, using a MDB subset of a continental genus‐level dataset (A. H. Thornhill, B. D. Mishler, N. Knerr, C. E. Gonzalez‐Orozco, C. M. Costion, D. M. Crayn, S. W. Laffan, and J. T. Miller. unpublished data). The MDB tree frog phylogeny was pruned to the MDB taxa from a two‐gene mitochondrial maximum likelihood phylogeny for the Australian–Papuan hylid radiation (Rosauer et al. 2009). The fish phylogenetic tree is a RAxML‐based phylogeny that used the mitochondrial cytochrome *b* gene derived from GenBank (P. Unmack, unpublished data).

### Diversity and endemism analyses of individual taxonomic groups

The spatial patterns of diversity for each of the five test groups were examined using multiple metrics: Taxon Richness (TR), Weighted Endemism (WE; Crisp et al. 2001), Phylogenetic Diversity (PD; Faith 1992), Phylogenetic Endemism (PE; Rosauer et al. 2009), Relative Phylogenetic Diversity (RPD; Mishler et al. 2014), and Relative Phylogenetic Endemism (RPE; Mishler et al. 2014). The calculation of RPD and RPE involves PD or PE measured on the actual tree divided by PD or PE measured on a comparison tree in which all branches are of equal length.

The statistical significance of PD and RPD was tested (two tailed test *α *= 0.05) using 999 trials against a null model where taxa are assigned randomly to grid cells but with the constraint that taxon richness, and the range size of each taxon, is held constant (Laffan and Crisp 2003; Mishler et al. 2014). This has the effect of making a random selection of the same number of terminals on the tree for a grid cell. The same randomization was used for CANAPE (categorical analysis of neo‐ and paleo‐endemism) (CANAPE; Mishler et al. 2014). CANAPE is a two‐step test, first testing for significantly high PE (one tailed test *α *= 0.05) and then for the cells passing step one a significance test of the RPE ratio (two tailed test *α *= 0.05). Grid cells which passed both of those tests were divided into four meaningful, nonoverlapping categories: neo‐endemism, paleo‐endemism, mixed‐endemism (i.e., high PE in step one at *α *= 0.05), and super‐endemism (high PE in step one at *α *= 0.01). The latter two types are places with a mixture of both neo‐ and paleo‐endemism, not dominated by either. Centers of neo‐endemism represent concentrations of rare short‐branched taxa, significantly low in the RPE ratio. Centers of paleo‐endemism represent concentrations of rare long‐branched taxa, significantly high in the RPE ratio.

### Fuzzy cluster analyses comparing patterns across taxonomic groups

Fuzzy clustering analyses, using Map Comparison Kit (MCK; Visser and de Nijs 2006) version 3.2, were applied to compare taxon groups pairwise for each of the observed TR, WE, PD, and PE diversity metrics across the MDB. We mapped dissimilarity values using a 200‐km‐diameter circle radius. We then used hierarchical clustering to group taxa with similar values for the different diversity metrics. Values approaching zero height on the resulting dendrogram suggest very similar spatial patterns across test groups, and height values closer to one suggest highly dissimilar groups.

### Phylogenetic beta‐diversity analyses of individual taxonomic groups

For each taxon group, a matrix of pairwise phylo‐jaccard dissimilarity scores between the assemblages in each pair of grid cells was used to identify clusters of phylogenetically similar regions, using biodiverse 0.18. The phylo‐jaccard index estimates the phylogenetic dissimilarity between two assemblages based on the fraction of shared phylogenetic branch lengths (Faith et al. 2009; Leprieur et al. 2012). We used the link average option for clustering, and the results were visualized using cluster tools in biodiverse.

### Diversity and endemism comparisons across taxonomic groups

Two measures were developed and applied to compare observed values of Taxon Richness (TR), Weighted Endemism (WE), Phylogenetic Diversity (PD), and Phylogenetic Endemism (PE) across all test groups: (1) mean for all grid cells represented and (2) mean for concordant grid cells. The second measure is used because not all organisms were present in all represented grid cells (i.e., fish are not present in areas without water), so we used only those grid cells with nonzero values for all test groups. To calculate both measures, first we standardized each test group to values from 0 to 1, where we subtracted the minimum diversity indices scores across all datasets divided by their range. In order to explore the potential effect of uneven sampling or distributions, that is, a bias due to restricted fishes, we also ran the analysis excluding the fish data.

Supporting information Tables S3–S6 have subsets of the data, R scripts, instructions, and examples of the steps needed to conduct the meta‐analyses.

## Results

### Diversity and endemism analyses of individual taxonomic groups

**Figure 2:**
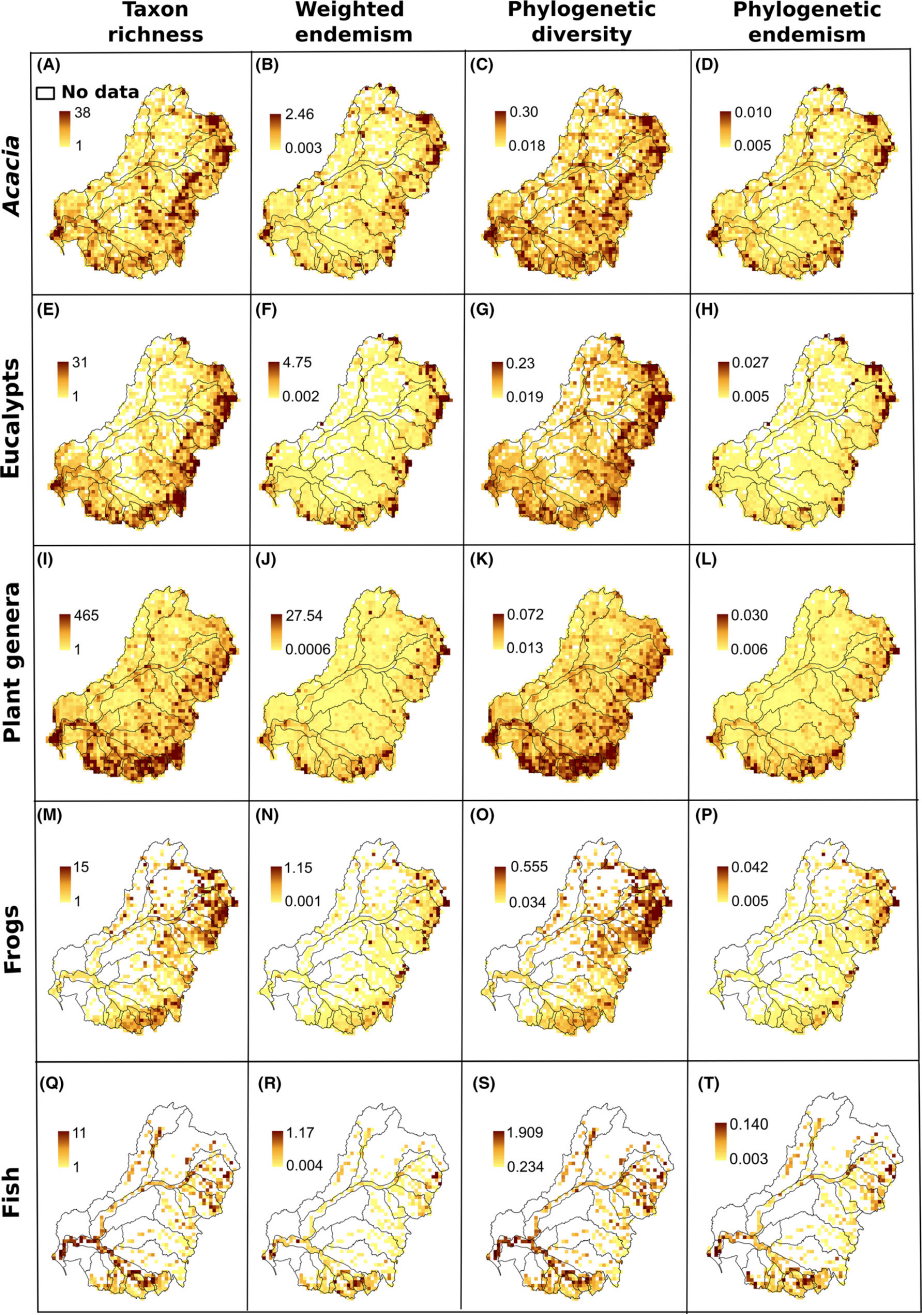
Observed patterns of diversity and endemism for five taxonomic groups in the MDB, southeastern Australia.

Maps of observed TR, WE, PD, and PE are presented for *Acacia* (Fig. 2A–D), eucalypts (Fig. 2E–H), plant genera (Fig. 2I–L), frogs (Fig. 2M–P), and fishes (Fig. 2Q–T). Overall, patterns of species, phylogenetic diversity, and endemism are not fully concordant across test groups. As expected, the plant groups display similar patterns but differ considerably from the animal groups. *Acacia* and eucalypts have highest TR along the foothills of the GDR, whereas the major areas of high WE and PE are on the mountainous areas either north or south along the GDR. Plant genus richness is highest in the southern part of the MDB with scattered areas of endemism mainly along the GDR. Areas of high TR and PD for frogs are in the northeastern part of the MDB with some minor areas of richness in the southeast. The main areas of high WE and PE for frogs are in the northeastern mountainous areas of the MDB. Fishes have two major areas of high TR and PD, one in the south along the Murray River and one in the northeastern tributaries of the Darling River. The main area of high WE for fish is in the lower mouth of the Murray River, with a small area of PE in the northeast and west of the upper Darling River.

**Figure 3:**
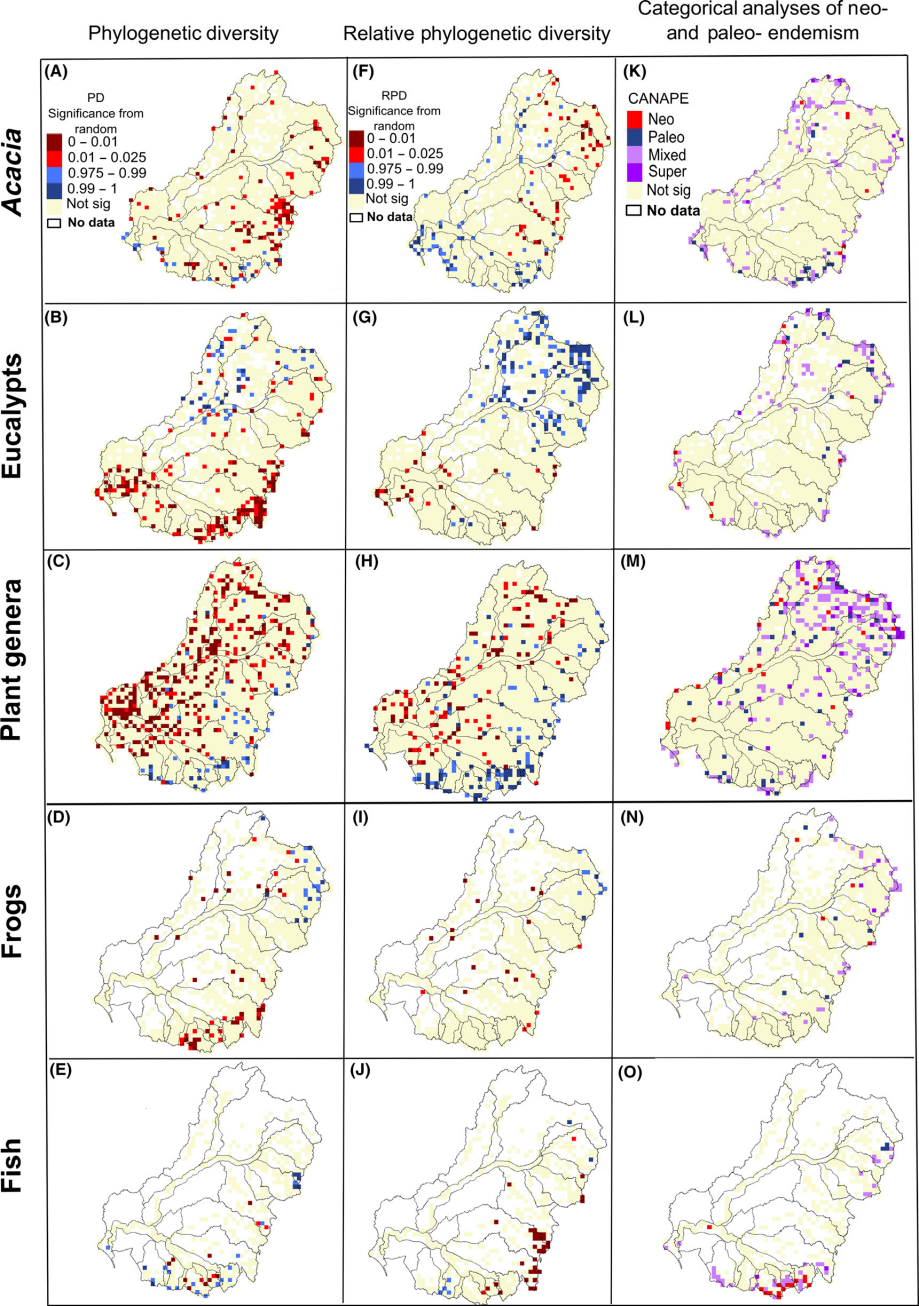
Centers of significantly high and low phylogenetic diversity (A–E), significantly high and low relative phylogenetic diversity (F–J), and centers of endemism identified by categorical analysis of neo‐ and paleo‐endemism (CANAPE; Mishler et al. 2014) (K–O) for all five taxonomic groups.

Centers of significant phylogenetic diversity and endemism for individual taxonomic groups were identified using the hypothesis tests of PD, RPD, and CANAPE (Fig. 3). Generally, we found a distinctive north–south division that potentially represents regions with lineages that are phylogenetically distinct and highly endemic (Fig. 3A–E). An east–west division is also apparent in the plants, which may correspond with the transition from low to high elevation.

Significance levels for PD show interesting contrasts among the taxon groups. *Acacia* and eucalypts show strikingly different patterns, with *Acacia* having significantly low‐PD sites (indicating phylogenetic clustering) in the north and significantly high‐PD sites (indicating phylogenetic over‐dispersion) in the south (Fig. 3A), with eucalypts having the opposite pattern (Fig. 3B). Plant genera show significantly low PD in the west and significantly high PD in the southeast (Fig. 3C). Frogs show significantly low PD in the southeast and significantly high PD in the northeast (Fig. 3D). Fish show a center of significantly high PD in the upper reaches of the Namoi Catchment, and mixed high and low PD in the south (Fig. 3E).

Significance levels for RPD show somewhat different patterns than for PD. *Acacia* and eucalypts show strikingly different patterns again, with *Acacia* having significantly low‐RPD sites (indicating a concentration of shorter branches than expected) in the east and significantly high‐RPD sites (indicating a concentration of longer branches than expected) across the south and in the west (Fig. 3F), while eucalypts have significantly low‐RPD sites in the southwest and significantly high‐RPD sites all across the north (Fig. 3G). Plant genera show significantly low RPD in the west and significantly high RPD in the south and east (Fig. 3H). Frogs show scattered grid cells with significantly low RPD throughout and a concentration of significantly high RPD in the northeast (Fig. 3I). Fish show a center of significantly high RPD in the south and significantly low RPD in the southeast (Fig. 3J).

The main centers of paleo‐endemism for *Acacia* are in the southeast of the MDB, whereas super‐endemic sites (those with a high concentration of both neo‐ and palaeo‐endemism) are either in the southeastern or northern regions (Fig. 3K). The main centers of paleo‐endemism and super‐endemism for eucalypts are in the north of the MDB, whereas centers of neo‐endemism are scattered in the northwest and southwest of the MDB (Fig. 3I). The main centers of paleo‐endemism and super‐endemism are in the northern part of the MDB for plant genera, but there is also a concentration along the southern edge (Fig. 3M). Frogs have mixed paleo‐endemism and neo‐endemism in the east part of the MDB (Fig. 3N). The main centers of paleo‐endemism and super‐endemism for fishes are in the northeast, while the main center of neo‐endemism is in the southeast (Fig. 3O).

### Fuzzy cluster analyses comparing patterns across taxonomic groups

**Figure 4:**
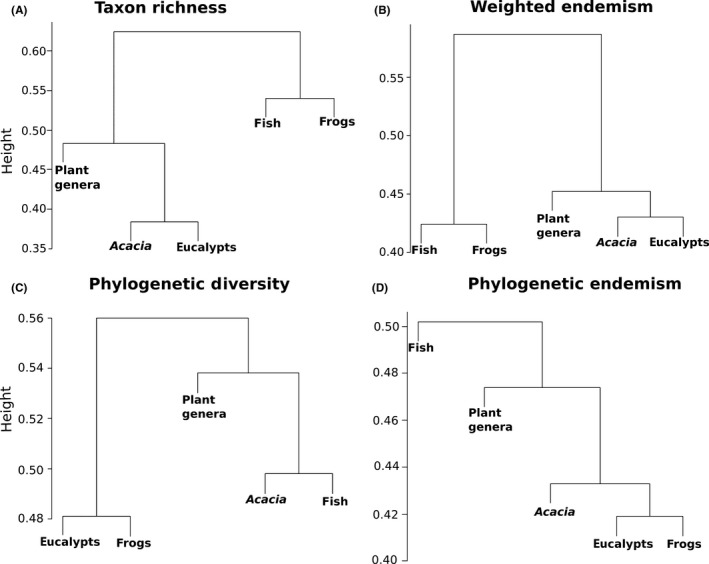
Fuzzy clustering analyses illustrating similarity in geographical patterns among taxonomic groups. A merger between groups closer to 1 means they are more dissimilar due to lower correlation in values for grid cells, whereas closer to 0 means more similar.

Similarities in observed spatial patterns of diversity among the five taxon groups are shown in Figure 4. As expected, taxon richness and weighed endemism cluster the five groups together in the same way (Fig. 4A and B); plants and animals are clustered in the two main branches of the dendrogram and *Acacia* and eucalypts are similar. In contrast, patterns in phylogenetic diversity and phylogenetic endemism cluster the five groups in different arrangements (Fig. 4C and D). Phylogenetic diversity clusters eucalypts with frogs and *Acacia* with fish (Fig. 4C), while phylogenetic endemism clusters eucalypts with frogs and then *Acacia* close to that pair (Fig. 4D).

### Phylogenetic beta‐diversity analyses of individual taxonomic groups

**Figure 5:**
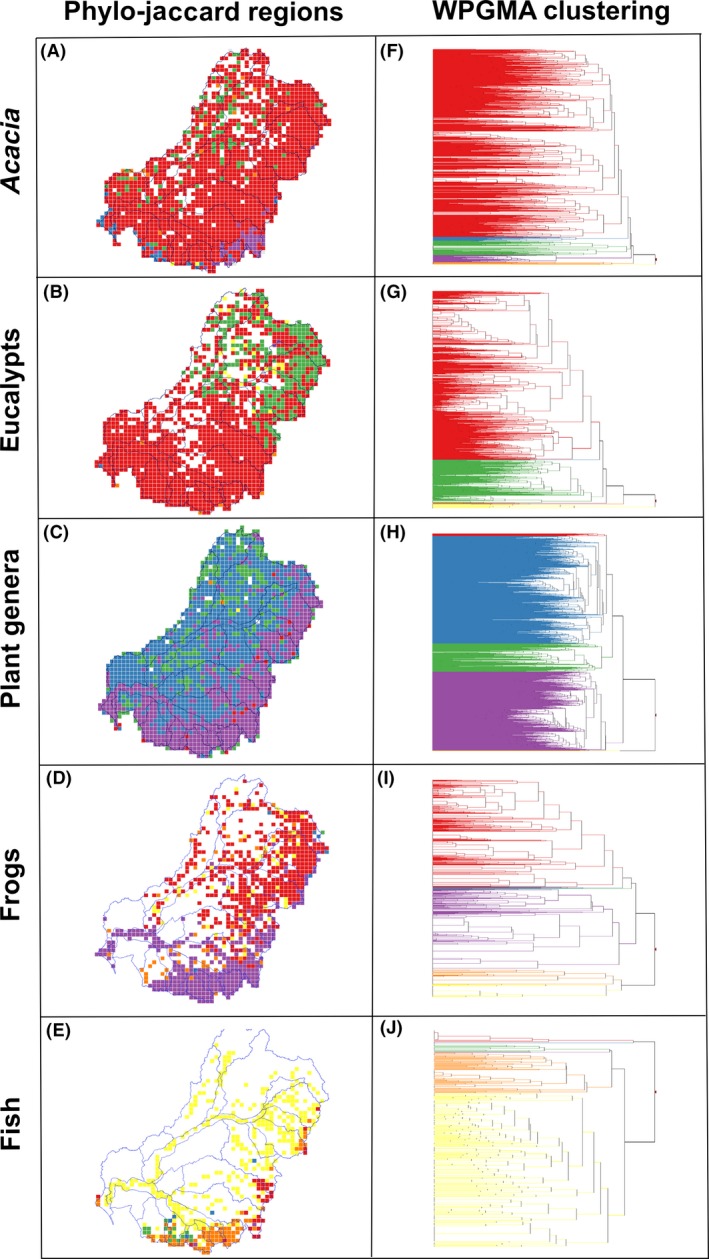
Phylogenetic beta‐diversity analyses of individual taxonomic groups in the MDB, southeastern Australia. The major phylo‐jaccard clusters are colored on the map (A–E) and dendrograms (F–J). These dendrograms show the similarity between grid cells in the portion of the phylogeny they share.

Patterns of phylogenetic beta‐diversity for all taxon groups are shown in Figure 5 (the different colors on the maps and dendrograms show the major clusters). There are major east–west and north–south breaks, depending on the test groups. Frogs (Fig. 5B and G) and eucalypts (Fig. 5D and I) have a similar north–south split. *Acacia* (Fig. 5A and F) and plant genera (Fig. 5C and H) have a similar east–west break which might be the result of elevation gradients and climate transition from the GDR into semi‐desert environments. Fishes (Fig. 5E and J) are distinctive, showing a major lowland cluster and a distinctive southeastern cluster.

### Diversity and endemism comparisons across taxonomic groups

**Figure 6:**
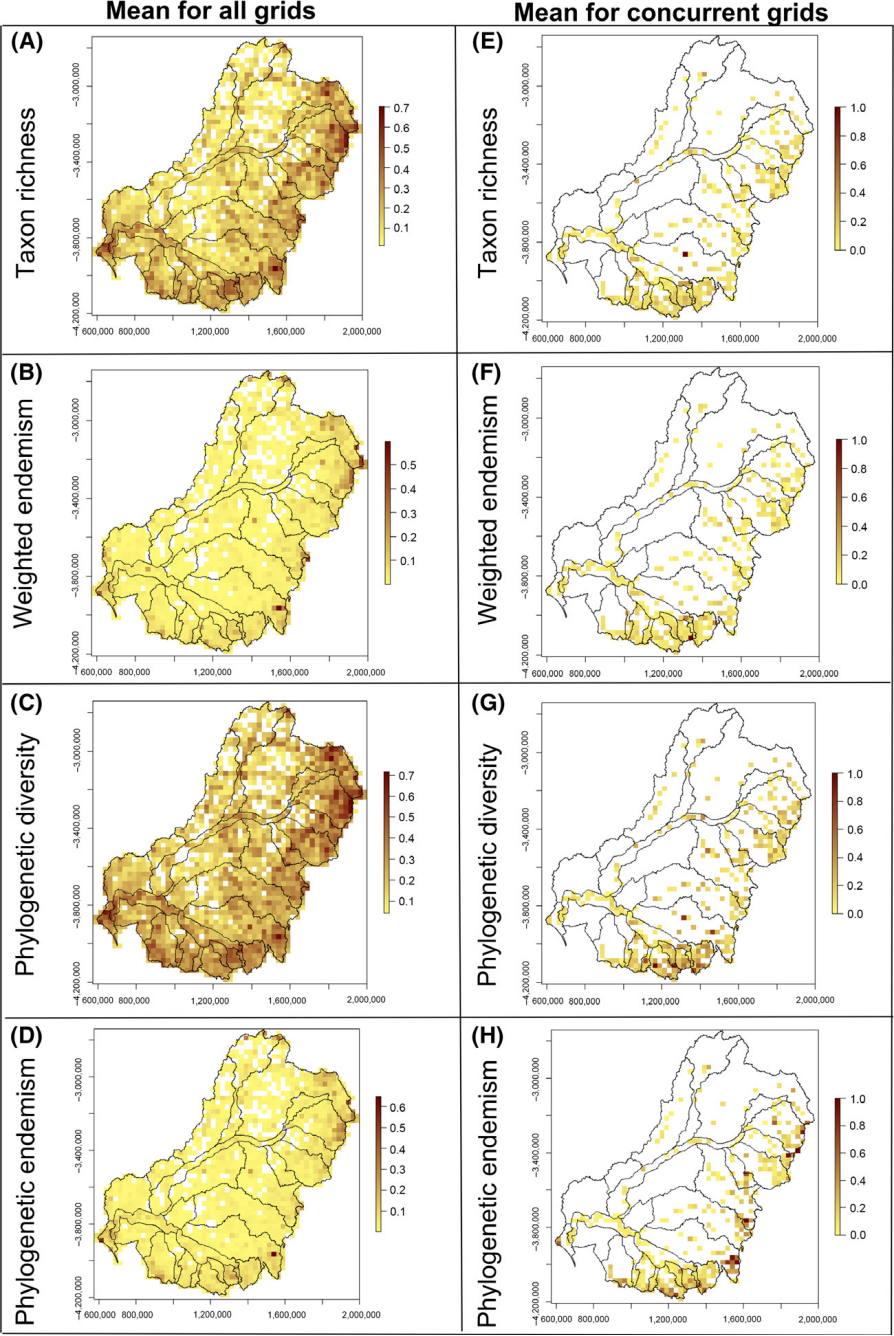
Maps comparing diversity and endemism across five taxonomic groups in the MDB, southeastern Australia, calculated using two spatial approaches: mean for all grids (left column) and mean for concordant grid cells (i.e., the mean for only those cells with all 5 groups present; right column). The different diversity metrics are as follows: taxon richness (A–E), weighted endemism (B–F), phylogenetic diversity (C–G), and phylogenetic endemism (D–H).

Comparing observed metrics of diversity and endemism across the five taxonomic groups allowed us to identify areas with generally high values (Fig. 6). The major concentration of high species and phylogenetic diversity across the test groups is located in the northeastern part of the MDB (Fig. 6A and C), whereas areas of high species and phylogenetic endemism are scattered along the highest mountainous regions of the GDR (Fig. 6B and D). Calculation of the mean for concurrent grid cells is limited by the distribution of test groups, especially when groups with limited distributions such as fish are included, but showed that the southeastern part of the MDB appears as the main concentration of diversity across all five test groups (Fig. 6E–H). After calculating the mean for concordant grid cells without the fish data, the number of grid cells under analysis increased substantially (Appendix S2; bottom row).

## Discussion

Our study explored how different diversity and endemism metrics (both taxonomic and phylogenetic) can be compared to conduct comprehensive assessments of biodiversity across multiple taxonomic groups. One of the major impediments to date has been that most biodiversity studies are limited to single taxon group analyses due to a lack of methods for comparing multiple groups of taxa (Ferrier 2002). Here, we have developed and applied a series of novel comparative approaches that improves our ability to simultaneously identify areas of common evolutionary history across multiple test groups (e.g., phylogenetic refugia) as well as areas containing unique histories of individual taxonomic groups.

Refugial hypotheses help to understand potential reasons for the existence of current phylo‐diversity areas. For example, hyper species‐rich areas such as southwest Western Australia, where there is adaptation to old and climatically buffered landscapes (Hopper 2009), have enhanced landscape stability creating multiple refugia across landscapes. On the other hand, in southern Australia, contraction and expansion of species ranges from the arid regions to more mesic environments were the result of Pleistocene climatic cycling which have generated major refugia (Byrne 2008). The MDB is an interesting region because it covers both semi‐arid and mesic environments. This is why locating centers of diversity and endemism across multiple taxon groups simultaneously is an interesting research exercise.

The major observed centers of high taxon diversity, weighted endemism, phylogenetic diversity, and phylogenetic endemism are located at the slopes of the Great Dividing Range in the eastern and southern portions of the MDB. *Acacia,* eucalypts, and plant genera have a similar pattern in these measures and differ from the animal groups to some extent (Fig. 2). However, the spatial location of significant centers of diversity and endemism detected in the hypothesis tests of PD, RPD, and CANAPE do not coincide with the observed centers, showing the importance of taking a statistical approach. Many places with high observed phylogenetic diversity are not significant as the PD value is what is predicted with that number of taxa. In fact, some places with high observed PD are significantly low, because they are rich in taxa and the PD is predicted under the null hypothesis to be even higher than that observed.

The major regions of significant phylogentic diversity and endemism across taxonomic groups in the MDB can be divided into three main sections: northern, southeastern, and western. The northern region experiences higher summer rainfall with drier winters, while the southern region has the opposite rainfall pattern. These are important biogeographical drivers of flora in Australia (González‐Orozco et al. 2014b). The southeastern and western distinction in significance patterns of RPD and CANAPE for *Acacia* and plant genera suggest the influence of climatic factors such as rainfall, which is to some extent related to elevation. In contrast, the north–south distinction in significance patterns observed in eucalypts, frogs, and fishes could be related to the geological history and geomorphology of the Murray and Darling river basins which are separated by the Lachlan fault.

The northern part of the MDB contains areas with significantly high‐PD, high‐RPD, and paleo‐endemism sites for eucalypts and frogs. This suggests phylogenetic over‐dispersion, with a significant concentration of long branches co‐occurring that are high in PE. A potential explanation for this is that the northern part of the MDB is subtropical in climate with higher rainfall at higher elevations which is ideal tree frog habitat. At the same time, the semi‐arid, but still subtropical, conditions on the lower floodplains of the Darling River might explain significant PD and PRD in the eucalypts. It may be a refugium, with some lineages (e.g., *Corymbia)* restricted to that transitional zone for a long time, occupying environments such as rainforest refugia along the great dividing ranges or pockets of humid habitat isolated among dry catchments. The significantly low PD for eucalypts and frogs in the extreme southeast of the MDB indicates phylogenetic clustering, (Webb et al. 2002) perhaps explained by evolutionarily conservative habitat preference of certain clades for the climate there with drier summers.

On the other hand, the southeastern part of the MDB contains areas with significantly high‐PD, high‐RPD, and paleo‐endemism sites for *Acacia* and plant genera. This suggests phylogenetic over‐dispersion (Webb et al. 2002), with a significant concentration of long branches co‐occurring that are high in PE. The eastern and southern parts of the MDB are more temperate and Mediterranean in climate, with cold temperatures and moderate rainfall. Variations in soil chemistry (e.g., variation in Ca‐Mg rates) maybe have led to certain pockets being preferred ecologically by different clades. The western semi‐arid floodplains show significantly low‐PD, low‐RPD, and neo‐endemism sites for plant genera. This suggests phylogenetic clustering, with a significant concentration of short branches co‐occurring that are high in PE. This could result from an evolutionary response to aridification; this region is predominantly low in elevation as well as having semi‐arid conditions. Certain major clades of plant groups may be adapted to desert‐like conditions in the west.

The fishes show a unique distribution of high‐PD, low‐PD, high‐RPD, and neo‐ and super‐endemism areas. Some upper catchments (in the far south and upper east) and the mouth of the Murray River have acted as refugia (i.e., isolated rock pools), collecting long branches over time. However, other upper catchments in the southeast have significant concentrations of short branches, some of restricted range, and thus appear to have played a role as centers for recent diversification.

The western semi‐arid floodplains are a significant region for plant genera because in that space, there are combinations of significant cases, where plant genera have low‐PD, low‐RPD, and neo‐endemism sites. It suggest that there are significant concentrations of short branches with close relatives that exclude each other and are significantly low in PE and at the same time are closely related. The potential reason of this pattern is an evolutionary response to aridification, because such region is predominantly low in elevation as well as having semi‐arid conditions on the western flood‐plains areas which may reflect deeper phylogenetic splits of the major clades of plant groups that adapted to desert‐like conditions in contrast to mesic conditions on the eastern part of the MDB.

The differences seen in the fuzzy clustering analyses for similarity patterns in different metrics highlights the fundamental differences between analyzing biodiversity using species measures versus phylogenetic measures. In specific cases like Figure 4C and D, we observed that biologically unrelated groups are clustered, such as here for both PD and PE of eucalypts and frogs. This is interesting because it indicates that they have correlated patterns in the amount of phylogenetic diversity and endemism in a given geographical area, which in turn suggests high geographical congruence of endemism hotspots. We observed that hotspots in PD and PE for both frogs and eucalypts occur in the same mountainous area in the northeast of the MDB. This may be owing to certain groups of eucalypts (*E. caliginosa, E. ligustrina, E. caleyi, E. amplifolia, E. terrica, E. prava)* and frogs (*Litoria lesueuri, L. fallax, L. verreauxii, L. latopalmata,* and *Cyclorana brevipes)* simultaneously evolving in environments with high elevations and subtropical rainfall. The other interesting result of the fuzzy clustering is the similarity between *Acacia* and fishes for PD. Both groups have congruent observed PD hotspots at the mouth of the Murray River (in the southwestern corner of the MDB) and appear clustered in Figure 4C, although only *Acacia* has significantly high PD in that area. A potential explanation for this congruence is the fact that the southwestern region of the MDB has low relief and high salinity soils. These aspects might reflect a historical relationship of organisms that did not necessarily evolve at the same time but are still affected independently by the same infertile soils and higher salinity (Bui et al. 2014).

We have demonstrated a new approach that allows identification of localities that have consistent values for both PD and PE across multiple taxonomic groups (Fig. 6). The summary maps comparing diversity and endemism across groups enhance the ability to identify such areas that cannot be identified using single taxonomic groups and therefore have new value for biodiversity studies. These summary maps have some current drawbacks, however. Limited spatial coverage in the distribution of biological collections is often the result of poor sampling effort (Schmidt‐Lebuhn et al. 2012).

There remain too few methods for incorporating phylogenies into conservation planning in general (Rolland et al. 2011), and even fewer methods to find areas with shared patterns of evolutionary diversity and endemism across multiple taxonomic groups. We have shown that it is possible to use multiple phylogenetic approaches to propose a conservation strategy for both shared and unique patterns of phylogenetic diversity and endemism, which are useful to promote effective conservation management (Davis et al. 2013).

There is not a full concurrence of phylodiversity centers across the studied taxon groups. However, we found several distinctive centers of phylogenetic diversity and endemism, often with more than two groups concordant for the same pattern. A further expansion of protected areas in the north part of the MDB is required. Such actions would potentially preserve unprotected unique evolutionary diversity in the MDB. A future extension of our approach could be a reserve network optimization algorithm such as Marxan or Zonation (Moilanen 2007; Ball et al. 2009) to identify the most valuable areas for conservation taking into account modeling uncertainties and environmental/phylogenetic data (Kujala et al. 2013; Rosauer et al. 2014). Owing to the rapid development of sequencing techniques and geo‐location of species occurrences at continental scale, it is likely that our approach could be applied and tested over the continental extent.

## Conflict of Interest

None declared.

## Data Accessibility

Example code with R scripts and subset of the data used in the manuscript, to run a meta‐analysis to assess biodiversity using phylogenetic methods across multiple taxonomic groups, are uploaded as online supporting information.

## Supporting information


**Appendix S1.** Phylogenies for each taxa (maximum likelihood RAxML).

**Appendix S2.** Comparison of the diversity patters using the mean for all grid cells analyses (upper panels) when fish data were excluded (lower panels) and the mean for concordant.

**Appendix S3.** Examples of meta‐analysis as a PDF with R scripts and instructions to run the basic calculations.

**Appendix S4.** Examples of meta‐analysis as an .rmd format to create a PDF or word document.

**Appendix S5.** Helper functions as an R script.

**Appendix S6.** Data subsets including acaciaex_grid.csv, fish_grid.csv, fishex_grid.csv, frogsex_grid.csv, plantgenex_grid.csv, knitbutton.png, pd_pairs_all.csv, pe_pairs_all.csv,sr_pairs_all.csv, we_pairs_all.csv, mdb.dbf, mdb.sbn, mdb.sbx, mdb.shp and mdb.shx.
