# Supplementary figures and images for: Assessing biodiversity and endemism using phylogenetic methods across multiple taxonomic groups
Source: Ecol Evol. 2015 Oct 22;5(22):5177–92. doi: 10.1002/ece3.1747 (PMC6102556; doi:10.1002/ece3.1747)

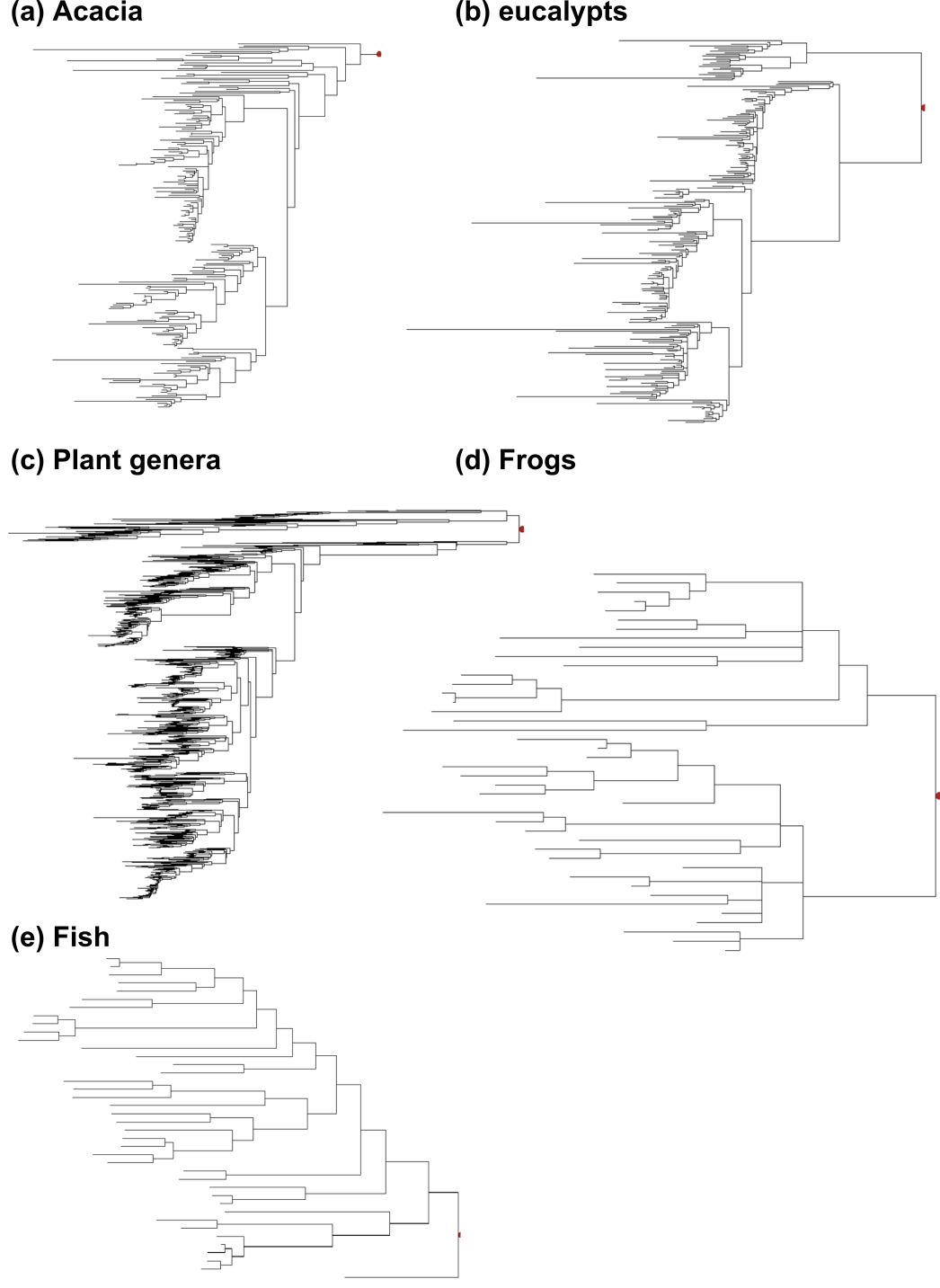


**Figure S**1**: Phylogenies used in our study.**

Supplement: Supplementary file 1 — Appendix S1. Phylogenies for each taxa (maximum likelihood RAxML). [file ECE3-5-5177-s001.doc]

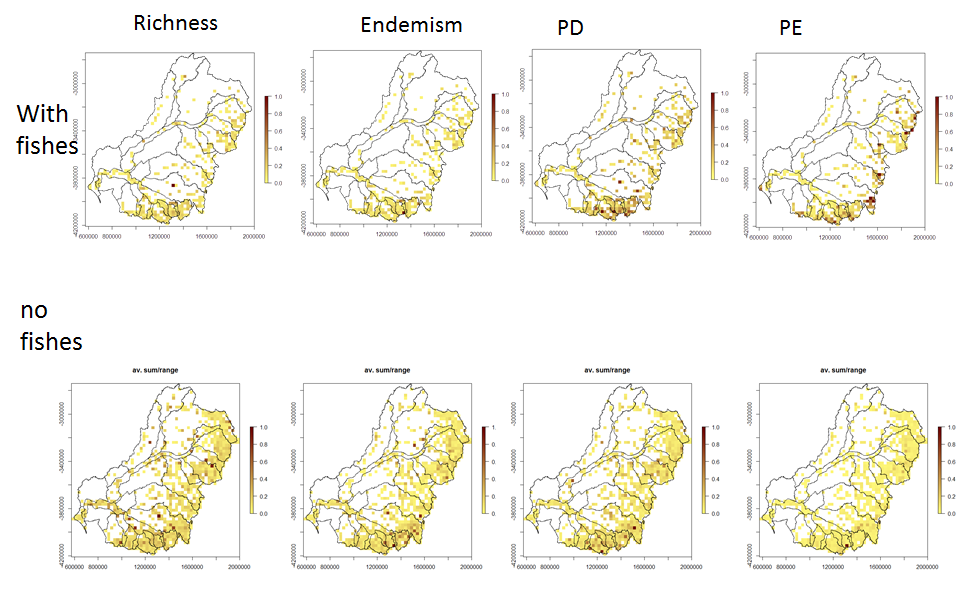


**Figure S2: Comparison of diversity standardized mean value when fish data were excluded.**

Supplement: Supplementary file 2 — Appendix S2. Comparison of the diversity patters using the mean for all grid cells analyses (upper panels) when fish data were excluded (lower panels) and the mean for concordant. [file ECE3-5-5177-s002.doc]

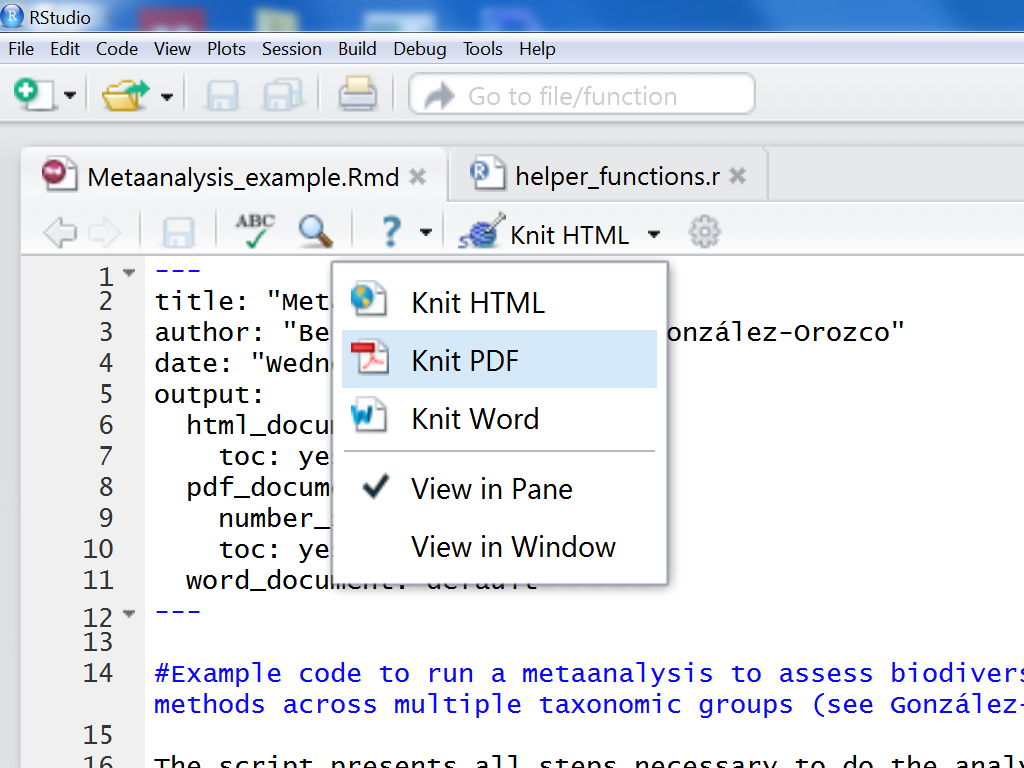

Supplement: Supplementary file 6 — Appendix S6. Data subsets including acaciaex_grid.csv, fish_grid.csv, fishex_grid.csv, frogsex_grid.csv, plantgenex_grid.csv, knitbutton.png, pd_pairs_all.csv, pe_pairs_all.csv,sr_pairs_all.csv, we_pairs_all.csv, mdb.dbf, mdb.sbn, mdb.sbx, mdb.shp and mdb.shx. [file ECE3-5-5177-s006.zip › Appendix_S6_knitbutton.png]
