# Supplementary material for: Assessing biodiversity and endemism using phylogenetic methods across multiple taxonomic groups
Source: Ecol Evol. 2015 Oct 22;5(22):5177–92. doi: 10.1002/ece3.1747 (PMC6102556; doi:10.1002/ece3.1747)

Meta_analysis_Examples

Bernd Gruber & Carlos E. González-Orozco

Wednesday, June 18, 2015

Contents

1. Example code to run a meta-analysis to assess biodiversity using phylogenetic methods across multiple taxonomic groups (see González-Orozco et al.) 1

2. Load data 2-3

3. DIVERSITY AND ENDEMISM ANALYSES OF SINGLE AND MULTIPLE TAXONOMIC GROUPS (Figure 2 and 6) 3-4

4. PHYLOGENETIC DIVERSITY AND ENDEMISM ANALYSES OF INDIVIDUAL TAXONOMIC GROUPS (Figure 3) 5

5. FUZZY CLUSTER ANALYSES OF INDIVIDUAL TAXONOMIC GROUPS (Figure 4) 5-6

6. PHYLOGENETIC BETA-DIVERSITY ANALYSES OF INDIVIDUAL GROUPS (Figure 5) 6-7

1. Example code to run a meta-analysis to assess biodiversity using phylogenetic methods across multiple taxonomic groups (see González-Orozco et al.)

The script presents all steps necessary to do the analysis of González-Orozco et al. (submitted) and consists of the following steps. Please note the full data set cannot be published at this state, due to copyright issues from external data providers, we will run the analysis on a subset of the data used in the manuscript. Therefore the results differ from the ones in the manuscript. The analysis comprises the following steps:

3. DIVERSITY AND ENDEMISM ANALYSES OF SINGLE AND MULTIPLE TAXONOMIC GROUPS (Figure 2 and 6) 3-4
4. PHYLOGENETIC DIVERSITY AND ENDEMISM ANALYSES OF INDIVIDUAL TAXONOMIC GROUPS (Figure 3) 5
5. FUZZY CLUSTER ANALYSES OF INDIVIDUAL TAXONOMIC GROUPS (Figure 4) 5-6
6. PHYLOGENETIC BETA-DIVERSITY ANALYSES OF INDIVIDUAL GROUPS (Figure 5) 6-7

To run the example script you need to have the following files and folders:

- Metaanalysis_example.Rmd
- helper_functions.r
- data folder (which contains the subfolders grid, mck and shape)

Simply execute the code provided in the gray boxes in the pdf file step by step or easier load the rmd file into rstudio and press the Knit button and select the document type you want to produce.

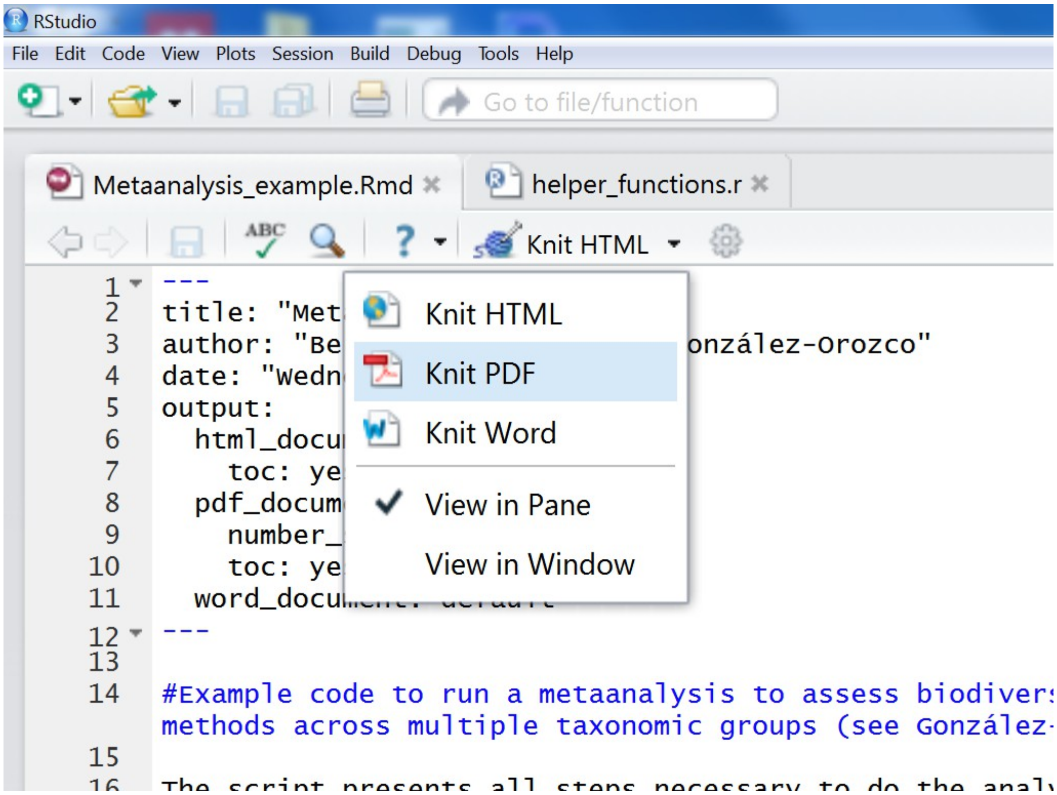

2. Load data

The data sets are gridded presence/absence data in Biodiverse format ([Laffan et al. 2010](#)). For a description how to run biodiverse please refer to the biodiverse home page http://purl.org/biodiverse. As mentioned above the provided data comprises a subset of the original data set only.

Laffan, S.W., Lubarsky, E. & Rosauer, D.F. (2010) Biodiverse, a tool for the spatial analysis of biological and related diversity. Ecography. Vol 33, 643-647 (Version 1.0)

```
library(raster)
library(SDMTools)
library(rgdal)
#set the working directory
```

```
setwd("d:/bernd/projects/crn/meta-analysis/")
#load some support functions
source("helper_functions.R")
```

3. DIVERSITY AND ENDEMISM ANALYSES OF SINGLE AND MULTIPLE TAXONOMIC GROUPS (Figure 2 and 5)

The code below creates maps of biodiversity patterns via the function complots. Basically we use the value at each coordinate of a certain column (e.g. Species richness="ENDC_RI", please refer to Biodiverse manuals or check the headings in the example data set, which options are available) and standardise it if specified. A combined plot summing all values of each species at each grid cell(e.g. combined species richness is also produced).

You can provide the following arguments to complots:

- ident = a string specifying which column in the data set is to be used e.g. "ENDC_RI"
- scale = a switch if the values should be scaled between 0 and 1
- ncols = number of colours to be used
- save = a switch if you want to save the plot in the working directory
- subsamp = a threhold that can be used to leave out data below a threshold
- complete = a switch: values for grid cells are only calculated if all species provide data for this cell (=FALSE) or at least on species provide data (=TRUE)

```
#create an empty list to load the data of each species
spec <- list()
#Find files of species data (needs to have grind in the name)
fn <- list.files("./data/grid/",pattern="grid")
#Load the data and store it in spec
for (i in 1:length(fn))
{
spec[[i]] <- read.csv(paste0("./data/grid/",fn[i]))
names(spec)[i] <- strsplit(fn[i],"_")[[1]][1]
}
#combine the data set into a single table specdat
specdat <- spec[[1]]
for (i in 2:length(fn))
specdat <- merge(specdat, spec[[i]][,-c(2:3)], by="Element", all=T)
#load shapefile (EsRI format)
map <-  readOGR("./data/shape/mdb.shp", "mdb", verbose=FALSE)
analysis <- c("ENDC_RI", "ENDC_WE" )# ,"PD_P", "PE_WE_P" ,"P_ENDC_WE",
"P_PD_P"
#run a loop over each specified biodiversty index
for (i in 1:length(analysis))
{
complots(ident = analysis[i], scale=T, ncols=100, save=T, subsamp=-1,
complete=F)
}
```

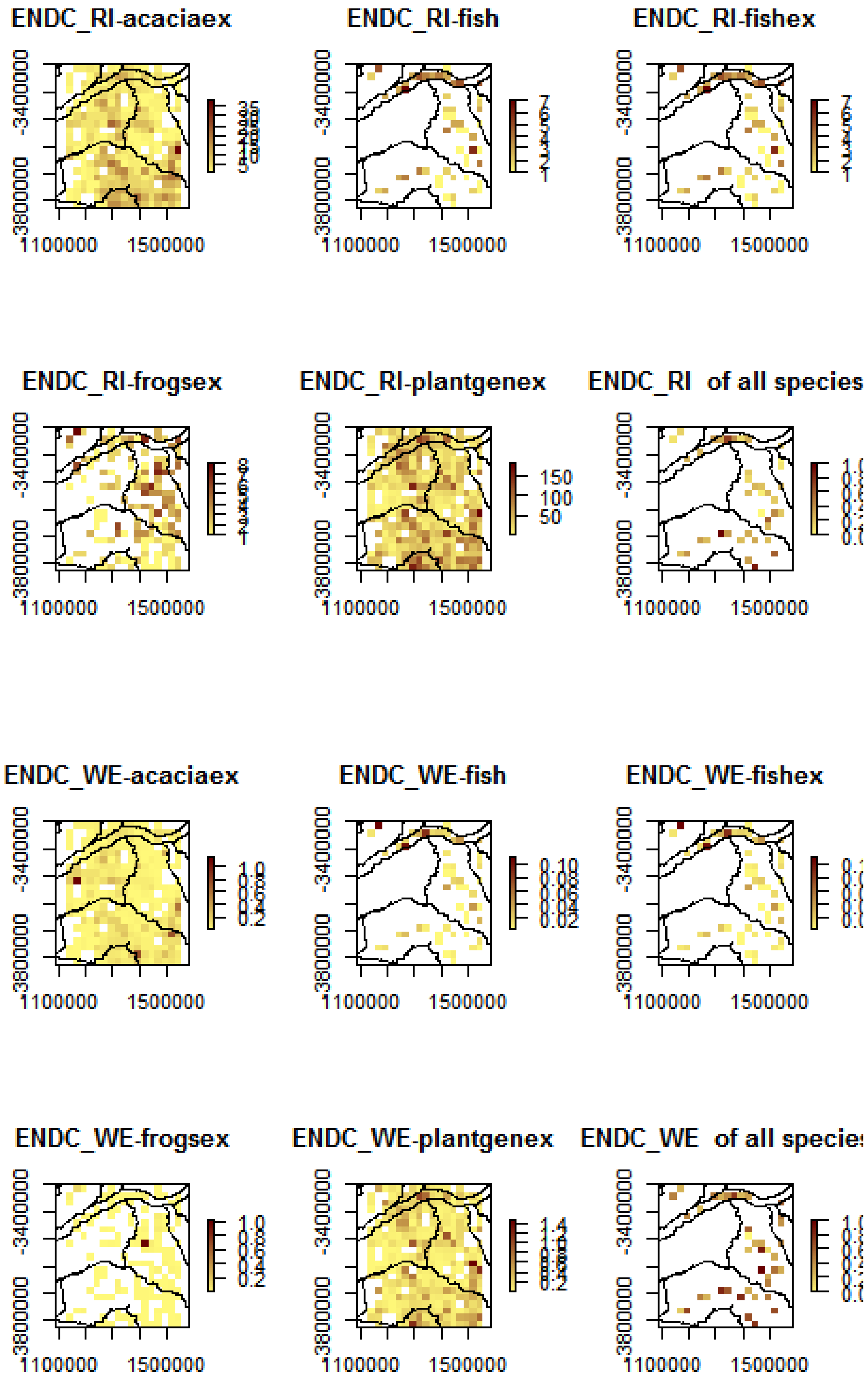
ENDC_RI-acaciaex
ENDC_RI-fish
ENDC_RI-fishex
ENDC_RI-frogsex
ENDC_RI-plantgenex
150
100
50
ENDC_RI of all species
ENDC_WE-acaciaex
ENDC_WE-fish
ENDC_WE-fishex
ENDC_WE-frogsex
ENDC_WE-plantgenex
ENDC_WE of all specie
-3400000
-3800000
1100000
1500000

4. PHYLOGENETIC DIVERSITY AND ENDEMISM ANALYSES OF INDIVIDUAL TAXONOMIC GROUPS (Figure 3)

For information how to create the CANAPE, PD and RPD plots and analyses of diversity and endemism please refer to the methods described in Mishler et al. (2014). (Below see sample of some plots as in Fig. 3 of the paper).

Mishler, B.D., Knerr, N., González-Orozco, C.E., Thornhill, A.H., Laffan, S.W., Miller, J.T. (2014) Phylogenetic measures of biodiversity and neo- and paleo-endemism in Australian Acacia. Nature Communications, 5, 4473

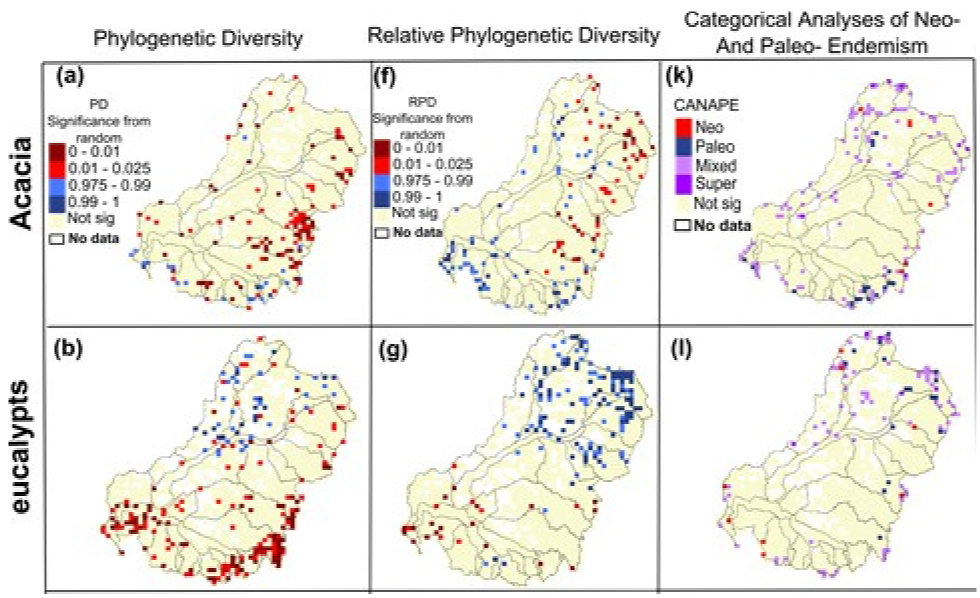

5. FUZZY CLUSTER ANALYSES OF INDIVIDUAL TAXONOMIC GROUPS (Figure 4)

The maps on biodiversity indices were used as input to the Map Comparison Kit (MCK; Visser & Nijs 2006) to apply a fuzzy clustering analysis to evaluate the similarity of these maps. The resulting dissimilarity values of the MCK were used within a cluster analysis.

Visser, H., de Nijs, T. (2006) The Map Comparison Kit. Environmental Modeling & Software, 21, 346-358.

```
#Example using Phylogenetic Endemism
srp <- read.csv("./data/mck/pe_pairs_all.csv")
m <- matrix(NA,nrow=5, ncol=5)
nn <-c("acacias", "eucs", "fishes", "frogs", "plants")

for (i in 1:10)
```

```
{
m[which(nn==srp$sp1[i]), which(nn==srp$sp2[i])] <- srp$pe[i]
m[which(nn==srp$sp2[i]), which(nn==srp$sp1[i])] <- srp$pe[i]
}
mm <- as.dist(1-m)
plot(hclust(mm ), labels=nn,ylim=c(0,1),main="Phylogenetic Endemism",
sub="",xlab=""  )
```

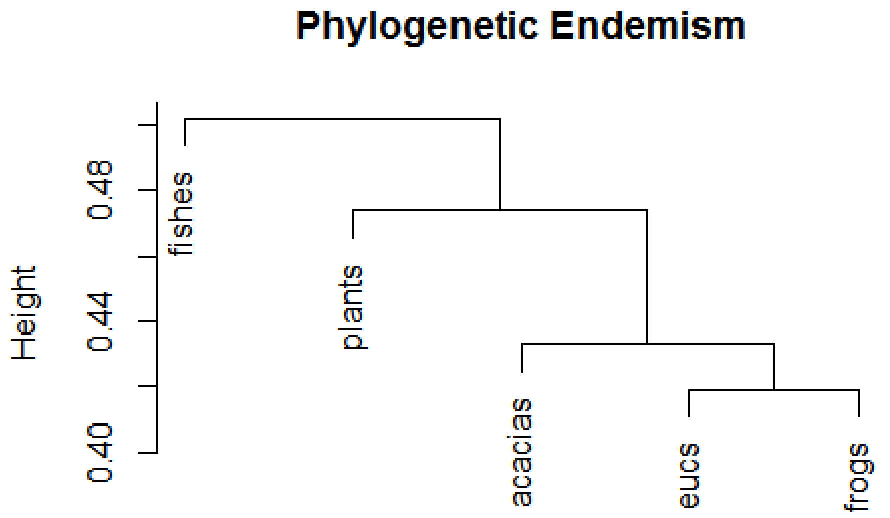

6. PHYLOGENETIC BETA-DIVERSITY ANALYSES OF INDIVIDUAL TAXONOMIC GROUPS (Figure 5)

For information how to create the phylo-jaccard maps refer to Laffan et al. 2010 in Biodiverse cluster analyses tools (Not scripted).

Laffan, S.W., Lubarsky, E. & Rosauer, D.F. (2010) Biodiverse, a tool for the spatial analysis of biological and related diversity. Ecography. Vol 33, 643-647 (Version 1.0)

Phylo Jaccard

Description: Jaccard phylogenetic dissimilarity between two sets of taxa, represented by spanning sets of branches

Subroutine: calc_phylo_jaccard

Index	*Index description*	*Grouping metric?*	*Minimum number of neighbour sets*	*Formula*
LO_JACCARD	Phylo Jaccard score	cluster metric	1	$= 1 - (A/(A + B + C))$ where A is the length of shared branches, and B and C are the length of branches found only in neighbour sets 1 and 2

Sample of phylo-jaccard index plots.

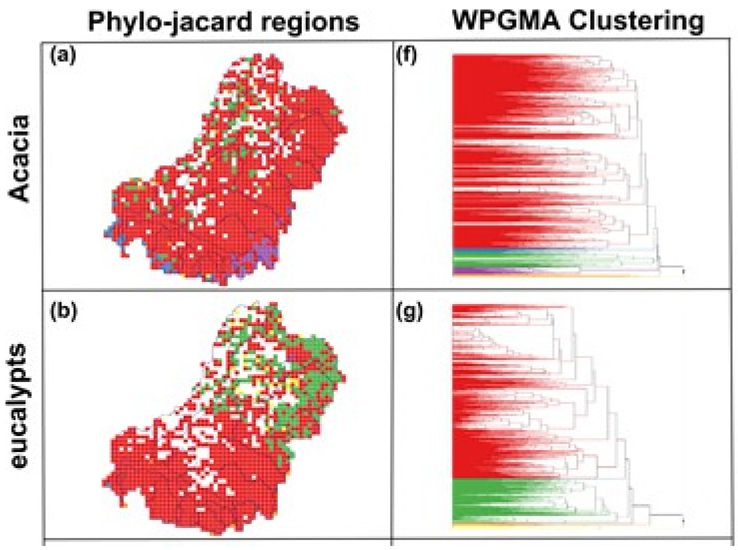

Supplement: Supplementary file 3 — Appendix S3. Examples of meta‐analysis as a PDF with R scripts and instructions to run the basic calculations. [file ECE3-5-5177-s003.pdf]
